# Surgical Outcomes of Syndesmotic Fixation of Ankle Fractures Using Syndesmotic Screws Versus Suture Button Devices

**DOI:** 10.7759/cureus.65051

**Published:** 2024-07-21

**Authors:** Roderick Kong, Shashidharan Viswanathan, Nima Razii, Shariff Hazarika

**Affiliations:** 1 Trauma and Orthopaedics, NHS Greater Glasgow and Clyde, Glasgow, GBR

**Keywords:** post-operative complication, fixation failure, syndesmotic instability, ankle fractures, ankle fracture management, syndesmotic screw fixation, suture button, fixation of syndesmosis

## Abstract

Introduction: Ankle fractures associated with disruption of the syndesmotic complex could potentially have poorer outcomes if missed or malreduced at the time of surgery. Favourable results have been reported for the suture button (SB) technique and may provide advantages over standard screw fixation of the syndesmosis, although this remains the gold standard method in many units.

Aim: To compare the outcomes of syndesmotic screws (SS) with SB fixation of the syndesmosis during ankle fracture fixation at a high-volume orthopaedic department of a Scotland trauma unit.

Method: A cross-sectional, retrospective study looking at ankle fracture fixations was undertaken at the Clyde Trauma Unit, Paisley. Relevant information was obtained from electronic patient records for 457 ankle fracture patients between August 2019 and February 2022 and followed up for six months. The digital patient archive system (PACS) was used for evaluating radiographs. Patients were divided into two groups depending on whether they had an SS or SB fixation of their syndesmosis. We focused on the surgical and radiological outcomes following syndesmotic fixation as no functional scores following surgery were conducted on the patients.

Result: Out of the entire study group, 26.3% (120/457 patients) required syndesmotic fixation. Within the syndesmotic fixation group, 70.8% (85/120 patients) underwent SS fixation, and 29.2% (35/120 patients) had an SB fixation. Both groups were statistically well-matched. Additionally, 21.1% (18/85) of SS fixation went on to have a second surgical procedure (four fixation failures, five planned removals, five for pain/stiffness, two infections, and two metalwork breakage/migration), whereas 8.6% (3/35) of the SB fixation group had a secondary procedure - two for fixation failures and one for infection.

Conclusion: We reported a higher incidence of associated syndesmotic injury in our series of 457 ankle fractures than previously described. There were significantly fewer sequelae in the SB group compared to the SS fixation group (P = 0.0464). Although we did not observe a statistically significant difference in the rate of reoperation (P = 0.1184), this is likely due to the small numbers in the SB group. Our study suggests that SB fixation may be associated with a lower rate of reoperation for post-op complications such as metalwork failure, pain, and stiffness (21.1% SS vs 8.6% SB). Regardless of the fixation method used, accurate reduction of the ankle mortice and syndesmosis is a key step to a successful surgical outcome.

## Introduction

Across the globe, the percentage of syndesmosis injuries following ankle fractures has been reported, ranging from 10% to 13% of all ankle fractures [1-7]. Within the UK, there is an incidence of 90,000 ankle fractures per year, and this translates to a significant number of encounters with such injuries in a high-volume orthopaedic department [8]. This number is predicted to rise further going into 2030 [9]. Identification and fixation of syndesmosis injuries is crucial in giving the patient the best chance of recovering ankle function and avoiding long-term symptoms of pain, stiffness, and overall patient dissatisfaction. The anatomy of the syndesmosis consists of the anterior inferior talofibular ligament (AITFL), posterior inferior talofibular ligament (PITFL), and the distal interosseus ligament, and these structures provide very strong stabilization to the ankle joint in motion by significantly limiting fibula external rotation and ankle mortise widening or rotation of the talus with ankle plantar and dorsiflexion [10-12]. Therefore, injuries to these structures can result in alteration of the ankle biomechanics when not adequately identified, reduced, and stabilized during surgery. Alterations in the ankle biomechanics following syndesmosis injuries include increased compressive stress within the tibia, increased likelihood of lateral dislocation of the distal fibula, and incongruence of the ankle articulating surfaces [12]. Subsequently, as the dynamic motion and relative joint position of the talus in relation to the ankle mortise changes, the distribution of the contact pressure across the articular surface becomes abnormal and can result in the development of joint arthritis [13]. Currently, several methods have been described in the literature as being used for syndesmotic fixation, namely, the use of syndesmotic screws (SS), bioabsorbable screws, and suture button (SB) fixation. Our department utilizes both SS and SB devices in syndesmosis fixation, and the aim is to compare the outcomes between these fixation methods in terms of surgical outcomes.

## Materials and methods

This study was designed as a cross-sectional, retrospective study. This study was exempt from the National Health Service (NHS) Research Ethics Committee review with Integrated Research Application System (IRAS) project ID 339006.

We reviewed ankle fractures surgically treated in the Clyde Sector Orthopaedic Service, which includes Royal Alexandra Hospital, Paisley, Scotland, as well as Inverclyde Royal Hospital, Greenock, Scotland. The cohort for the study was gathered by running a custom query through Bluespier™ (Droitwitch, England) theatre management software. All ankle fracture fixations which were done in both hospitals between the period of August 2019 and February 2022 were included in the study. The main inclusion criterion for this study was patients who sustained ankle fractures fitting into the Lauge-Hansen classification for ankle fractures. The Lauge-Hansen classification was pioneered by Danish physician, Neils Lauge-Hansen in 1942 through cadaveric studies by applying external force to amputated ankles until a fracture was created and the patterns studied. This resulted in four categories of mechanism injury, namely, supination external rotation (SER), pronation external rotation (PER), supination adduction (SAD), and pronation abduction (PAB). Furthermore, we included patients who had single, isolated ankle fractures and subsequently had definitive internal fixation only. For our exclusion criteria, we excluded patients whose fractures do not fit into the mechanism of injury described by Lauge-Hansen meaning that pilon fractures and distal tibial fractures which were listed as ankle fracture fixation on the trauma theatre management software were excluded. Patients who had external fixation only for the entirety of surgical management were excluded. We also excluded patients who sustained polytrauma with more than one significant injury requiring surgical fixation, as well as bilateral ankle injuries. In addition, we excluded patients who had delayed presentation of their fractures and any fractures deemed pathological such as pre-existing abnormality in the bone architecture due to malignancy or infection.

Patients' demographic data, past medical history, and perioperative and postoperative care were collected by reviewing electronic medical records, and any imaging was reviewed using patient archive system (PACS). Demographic data collected includes age, gender, and BMI. Medical records were reviewed to note the past medical history of peripheral vascular disease, diabetes mellitus, and osteoporosis. A history of smoking, alcohol consumption, anticoagulation, and steroid use was noted as well. Furthermore, the study looked at the mechanism of injury (high energy vs low energy); the type of fracture based on the Lauge-Hansen classification, as mentioned in the inclusion criteria earlier, if it was an open or closed injury; and the extent of dislocation of the joint.

Information regarding the patient’s pre-operative management including the adequacy of reduction and immobilization and any initial external fixation to achieve reduction was noted. Some patients underwent further three-dimensional imaging pre-operative planning. Patients had either SS or SB devices to fix the syndesmosis depending on surgeons’ choice and familiarity with the surgical technique.

Data collected included the operative records, including time to definitive fixation, level of primary surgeon, number of incisions used, surgical approaches used, plastic surgery input if any, and the fixation devices used. 

Post-operative data regarding the length of patient inpatient stay, weight-bearing status at discharge, type of venous thromboembolism (VTE) prophylaxis, length of follow-up, sequelae, and any further surgeries.

Patients were followed up in the clinic for any complications following surgery, including fixation failure and displacement of the syndesmosis, fixation device breakage or migration, post-operative symptoms affecting function such as pain and stiffness, and infection. The assessment of the function was purely based on patients’ subjective reporting of any symptoms of pain and stiffness post-surgery which were obtained from clinical letters, and no formal functional score was done given the limited resources in the follow-up clinical setting. Reasons for patients who underwent further surgery were investigated.

The surgical technique included fixation of fibular fracture and any associated medial malleolus fracture. The method of fixation of the fibula was not controlled in this study as it includes using a 1/3 tubular plate, distal fibula locking plate, and with or without an interfragmentary screw. Furthermore, the method of medial malleolus fracture fixation was not controlled and can include the use of two parallel cancellous screws or plate fixation in the buttress plating mode. This study focussed on restoration of ankle mortise following adequate fixation of both malleoli, restoration of length, and alignment & rotation of the fibula.

**Figure 1:**
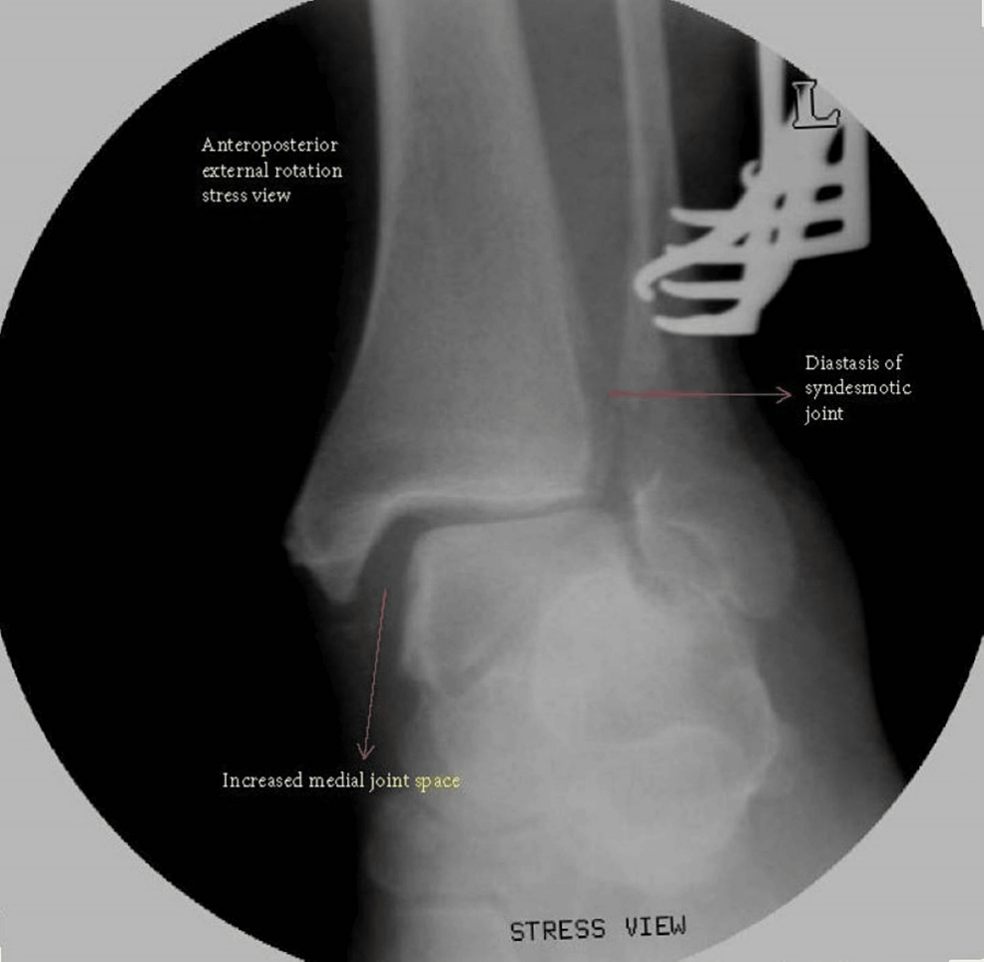
Intraoperative external rotation stress view demonstrating syndesmotic injury

Following malleolar fracture fixation, the integrity of the syndesmosis is assessed using a bone hook applied on the distal fibula and lateral force applied with external rotation of the talus and under image intensifier. If syndesmosis injury is identified, reduction of the syndesmosis is performed, and fixation of the syndesmosis is carried out. Figure 1 shows an example of syndesmotic injury demonstrated on stress views with an image intensifier intraoperatively.

Syndemosis fixation is done by initially drilling a hole from the lateral distal fibula through to the lateral cortex of the tibia just proximal to the inferior tibiofibular joint, at 30 degrees from posterior to anterior, parallel to the tibial plafond, and with the ankle joint in neutral.

Once the cortices have been prepared, the fixation device used here can be a syndesmotic screw which is a 3.5 mm cortical screw passed through the tract prepared by the drilling earlier. One or two screws were used passing through either three or all four cortices.

**Figure 2:**
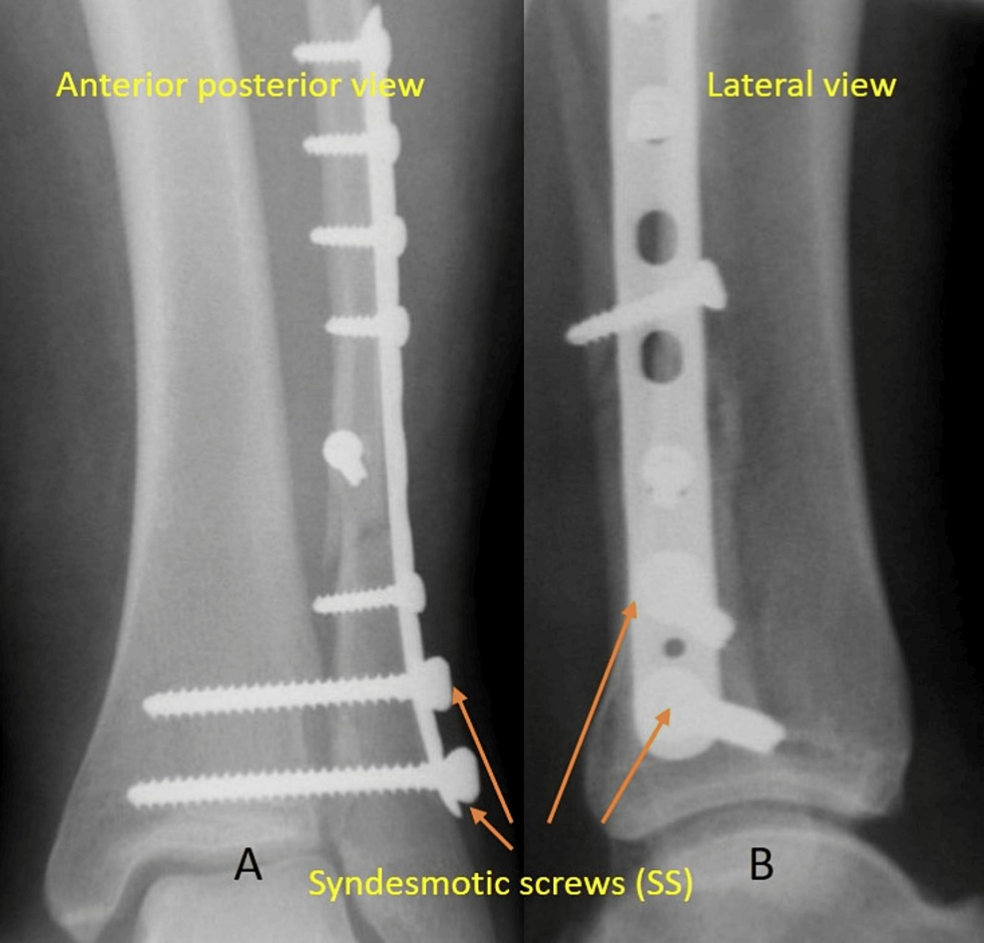
Intraoperative imaging post syndesmotic fixation with syndesmotic screws A & B: AP and lateral views, respectively, following suture button fixation

Alternatively, an SB device can be used which, in our department, is the TightRope ™ by Arthrex (Naples, Florida). Either one or two were used, depending on the surgeon’s choice. The use of the SB device by default will have to go through all four cortices from the lateral distal fibula to the medial tibia to work [14]. Figure 2 shows the appearance of the syndesmotic fixation using SS. 

**Figure 3:**
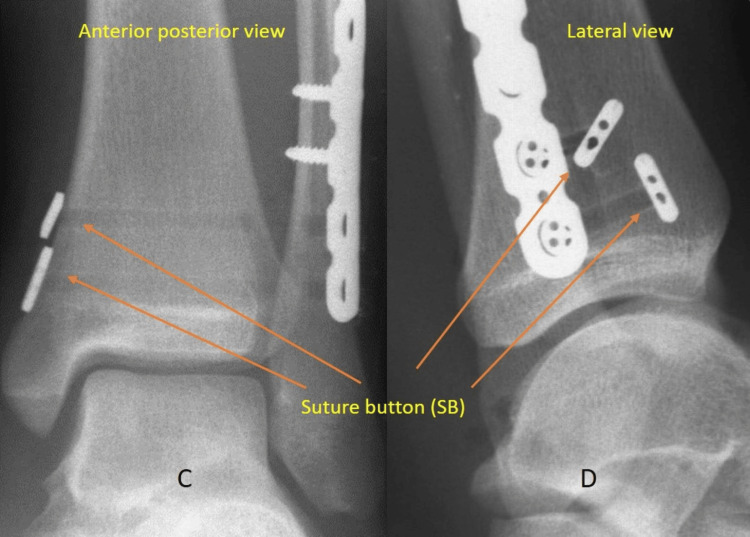
Intraoperative imaging post syndesmotic fixation with suture button C & D: AP and lateral views, respectively, following syndesmotic screw fixation

Figure 3 shows the intraoperative appearances of syndesmotic fixation using the SB device.

## Results

**Figure 4:**
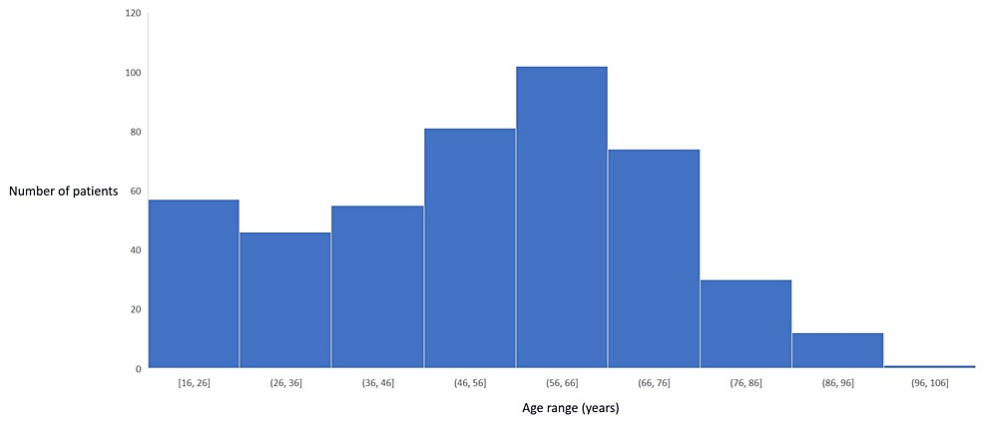
Age distribution of patients who underwent ankle open reduction & internal fixation

In total, we gathered a total of 457 patients in this cohort for the study over a 36-month period who underwent ankle fracture fixations. The median age was 56 years old ranging from 16 years to 97 years old. Overall, there was a bimodal distribution with a peak in the 16-26-year-old group and a second peak in the 56-66-year-old group. Figure 4 shows the age distribution of patients who underwent ankle fracture fixation.

**Figure 5:**
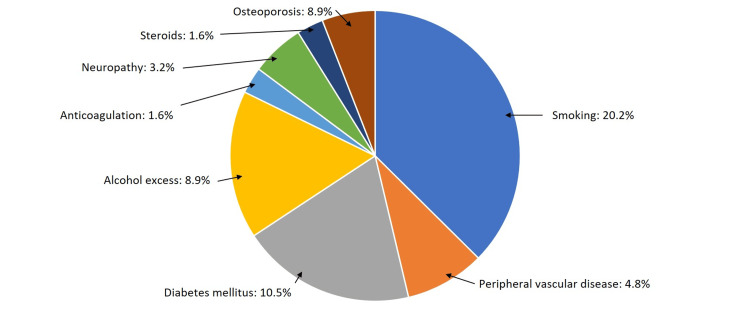
Distribution of all patients based on co-morbidities

The top four most common comorbidities in the cohort were smoking (20.2%), diabetes mellitus (10.5%), alcohol excess (8.9%), and osteoporosis (8.9%), as shown in Figure 5, which also includes the distribution of the other relevant comorbidities.

**Figure 6:**
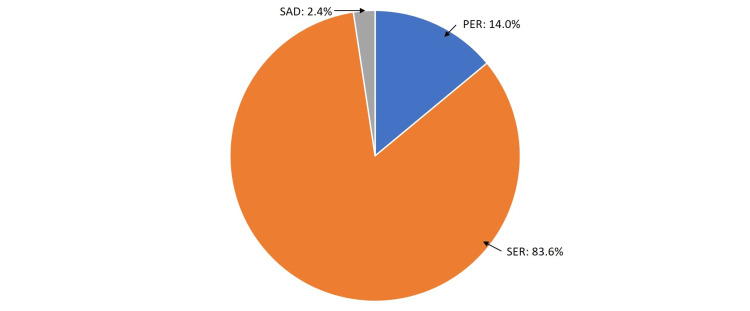
Distribution of ankle fractures based on the mechanism of injury using the Lauge-Hansen classification PER: Pronation-external rotation; SER: Supination-external rotation; SAD: Supination adduction

Out of these 457 patients, 83.6% (382 patients) were SER injuries, 14.0% (64 patients) were PER injuries, and 2.4% (11 patients) were supination adduction injuries with significant medial malleolar fracture warranting surgical fixation. Figure 6 shows the distribution of ankle fractures based on the mechanism of injury using the Lauge-Hansen classification.

Overall, out of the 457 patients in this study group, 26.3% (120 patients) had syndesmotic fixation done. In this group, 51% were SER injuries, and 49% were PER injuries.

**Figure 7:**
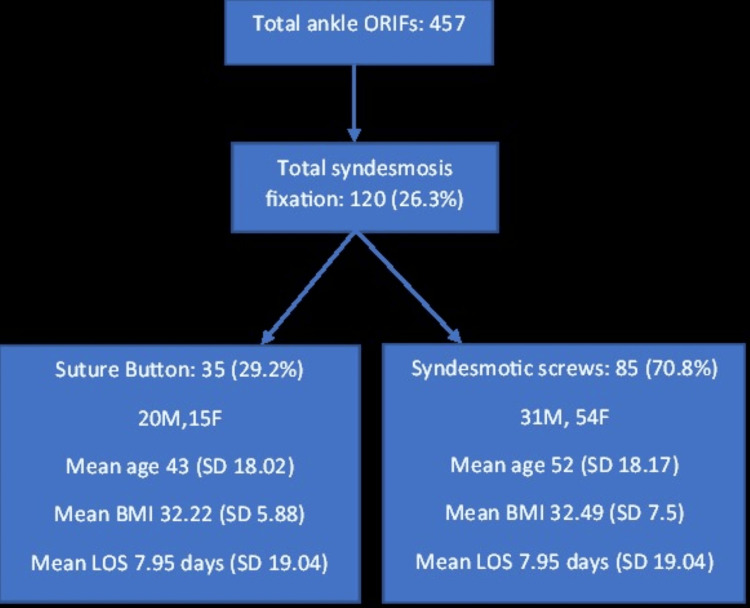
Breakdown of syndesmotic fixation among the ankle fixations SD: Standard deviation; BMI: Body mass index; LOS: Length of stay

From this group, 70.8% (85 patients) were fixed using SS, and 29.2% (35 patients) were fixed using the SB device. Figure 7 shows the breakdown of patients who had syndesmotic fixation using SS and SB along with the mean age, BMI, and length of stay in the hospital. 

Having gathered information following an investigation into all the patient factors, as well as the perioperative and post-operative management, we compared both the SS and SB groups based on those criteria to see how matched the groups were. We utilized the unpaired t-test and Fischer’s exact test when comparing these two groups. The results show that the only real statistical difference was that there were more men who had SB fixation and more women who had syndesmotic screw fixation (P = 0.0439, Fischer’s exact test) and that the SB group had significantly younger patients (mean age: 43 years old) as opposed to the SS group (mean age: 52 years old) (P = 0.0201, unpaired t-test). Otherwise, both groups were statistically well-matched within the limitations of a non-randomized selection.

Overall, there were statistically significant differences in the complications between the SB and SS groups with the SB group having lesser complications (P = 0.0464, Fischer’s exact test). However, not all these patients underwent further surgery.

**Figure 8:**
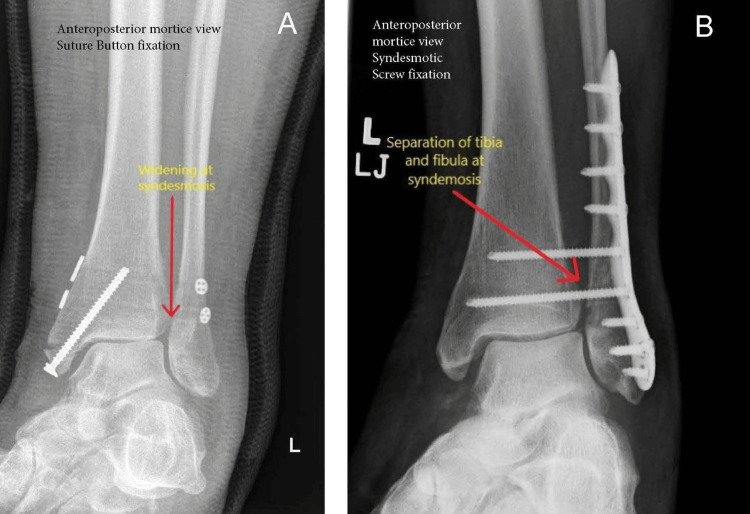
Plain radiograph showing widening of the increased separation of the tibia and fibula following fixation with the suture button device (A) & syndesmotic screws (B), indicating failure of fixation

**Figure 9:**
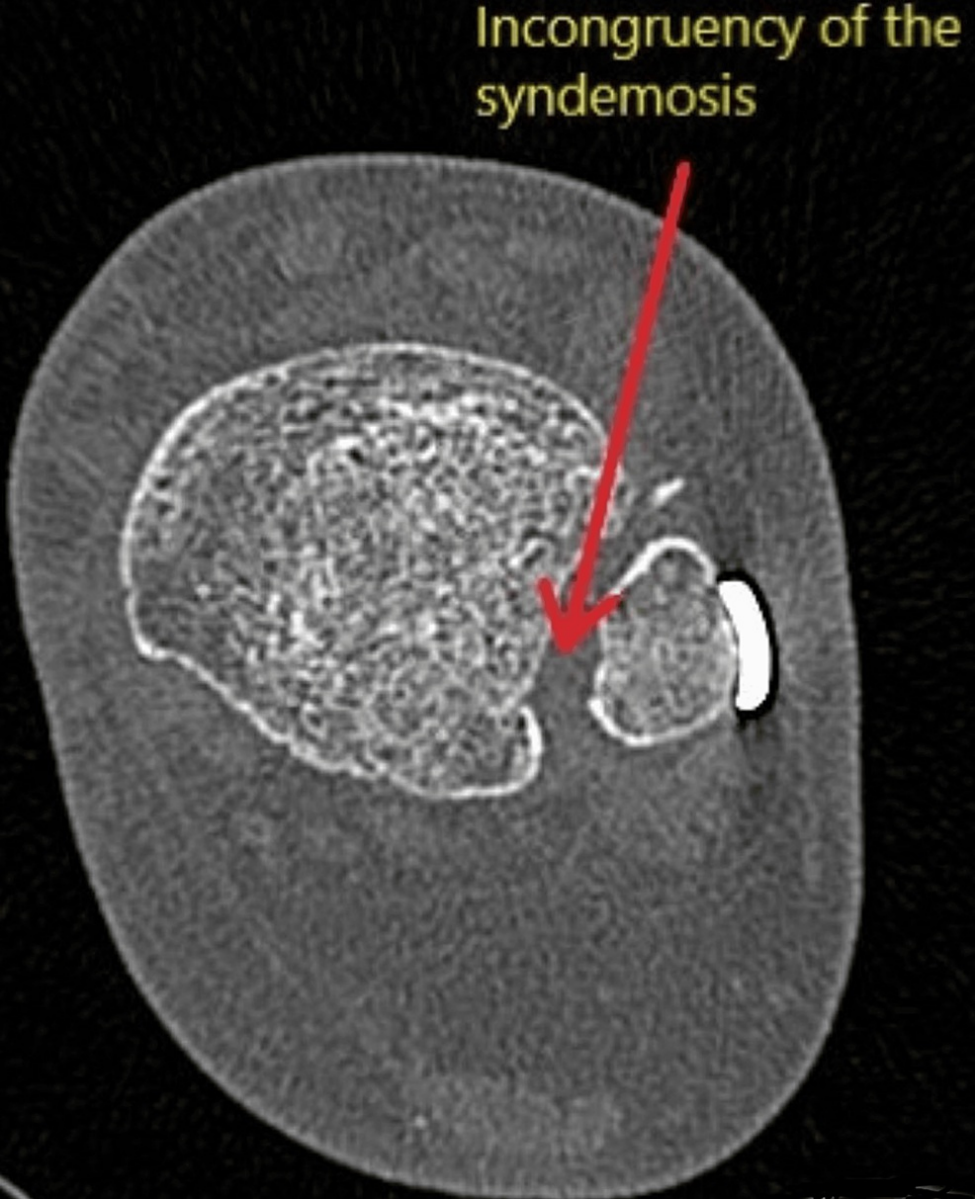
Non-contrast computed tomography (CT) scan of the ankle fixed with the suture button device showing incongruency of the syndesmosis

Additionally, 21.1% (18 patients) underwent a second surgical procedure in the SS group. Out of these 18 patients, four patients required revision fixation, and 14 required removal of metalwork for a combination of infection, metalwork breakage or migration, and post-operative symptoms. For the patients who did not end up with further surgery, one patient experienced delayed union, two experienced temporary symptomatic syndesmosis screw breakage, and one patient experienced a wound infection not requiring further surgery. Figure 8 shows examples of plain radiographs demonstrating the failure of fixation using the SB device and using SS. Figure 9 shows the computed tomography (CT) image slice of the syndesmosis and non-congruency, indicating malreduction and fixation failure using the SB device.

Moreover, 8.6% (three patients) underwent a second surgical procedure in the SB group. Two patients required revision fixation, and one required removal of metalwork due to infection.

Whilst the rates of re-operation between the two groups were not statistically significantly different (P = 0.1184, Fisher’s exact test), they could be considered clinically significant. The rate of re-operation was 8.6% in the SB group, whilst it was 21.2% in the SS group which is almost three times higher.

## Discussion

Despite the lack of statistically significant difference in the re-operation rate, we must keep in mind that we are focusing on surgeon-centred outcomes of complication and reoperation rates. For the patient experience, the significantly lower rate of sequelae means not being put under any undue distress due to post-surgical complications of any kind which can translate to shorter time to rehabilitation and recovery which are important determinants in the overall outcome of ankle function. Although the syndesmosis is described as a very strong ankle stabilizer, there is still a degree of dynamism in the ligamentous complex which allow for mobility of the ankle joint. The use of SB devices can potentially offer stability for healing but enough movement for restoring ankle biomechanics and function, without the need for planned screw removals or metalwork issues as observed in the SS group.

The fixation failures and post-surgical complications are also heavily influenced by patient factors. The population group operated on does have a large percentage of patients with lifestyle choices such as smoking and alcohol excess which contribute to weaker bone architecture due to altered bone metabolic physiology and, hence, poorer surgical outcomes and fixation failure even with appropriate reduction and fixation of the syndesmosis. Furthermore, the prevalence of co-morbidities such as higher BMI, diabetes mellitus, and osteoporosis can contribute to adverse outcomes regardless of the fixation device used for the stabilization of the syndesmosis.

Although the use of SB devices over SS is encouraging, we acknowledge that there are limitations to this study. Firstly, the fact that there was no statistically significant difference in the rate of re-operation between both groups is likely due to the small number in the SB group leading to a type II error. Furthermore, the patient selection was not randomized. As mentioned earlier, another limitation was that we looked at surgeon-centred outcomes and not patient-centred outcomes. The patients were also not controlled for age and gender and the statistical analysis did demonstrate significantly more male patients who had SB fixation for the syndesmosis, as well as younger patients in the SB group which is also a limitation of the study.

When doing a comparative literature search, there is evidence from at least three meta-analyses in the last five years which demonstrate that functional outcomes using SB were better for patients [15-17]. Xu et al. [15] found that, across 389 studies, there was no significant difference in malreduction of the fracture in both the SS and SB groups. The functional scores utilized across these studies were the American Orthopaedic Foot & Ankle Society (AOFAS) score, the Olerud-Molander ankle score (OMAS), and the EuroQol-5 Domain (EQ-5D) score. The SB group showed statistically better function when the AOFAS and OMAS were used. Xu et al. [16] included 12 clinical trials and overall did not find a statistical difference in satisfactory syndesmosis reduction based on radiological appearance or clinical outcomes in both the SS and SB groups. The SB group did have improved clinical outcomes. When the study focuses on the surgical outcomes of good reduction, this factor is surgeon-dependent and hence does not rely on the device being used. From the patient’s perspective, however, the SB group patients appear to achieve better function post-operatively, and this can put more weight in favour of choosing the SB device for syndesmosis fixation. Two long-term follow-up randomized control trials found that both patient and surgical outcomes were quite similar [18,19 ]. Altmeppen et al. [18] demonstrated in a randomized clinical trial that patients who underwent SB device fixation for syndesmosis injury had a faster return to activity, especially in high functional demand patients such as athletes. However, when looked at over a 10-year period, the overall outcome in clinical scores was similar for both SS and SB groups. Similarly, Lehtola et al. [19], whose study followed patients over a six-year period, demonstrated no significant difference in functional outcomes of both the SS and SB groups.

The decision whether to use an SS or an SB device based on the literature review generally is up to the surgeon’s preference and experience. In the context of high functional demand patients who require a quicker return to activity, an argument can be made for using the SB device. Otherwise, in general, the outcomes both surgically and functionally are similar in the long term, and any benefits of one method over the other only appear to be in the short term.

## Conclusions

There is an associated learning curve with using the SB device for syndesmotic stabilisation, but in our unit, it has been successfully used by surgeons of varying levels of experience, and we have observed a lower rate of fixation failure and reoperation rate when compared with SS. However, the choice of fixation does not substitute good surgical acumen in terms of identifying and adequately managing syndesmosis injuries intra-operatively. We advocate adequate intraoperative exploration and visualisation of the syndesmosis where there is suspicion of an injury, before a direct reduction and stabilisation. In addition, we reported a comparatively higher incidence of syndesmotic injuries in our study, highlighting the importance of a higher index of suspicion pre-operatively.
